# A blended psychosocial support program for partners of patients with amyotrophic lateral sclerosis and progressive muscular atrophy: protocol of a randomized controlled trial

**DOI:** 10.1186/s40359-018-0232-5

**Published:** 2018-05-02

**Authors:** Jessica de Wit, Anita Beelen, Constance H. C. Drossaert, Ruud Kolijn, Leonard H. van den Berg, Johanna M. A. Visser-Meily, Carin D. Schröder

**Affiliations:** 1 Center of Excellence in Rehabilitation Medicine, Brain Center Rudolf Magnus, University Medical Center Utrecht, Utrecht University and De Hoogstraat Rehabilitation, Utrecht, the Netherlands; 2 Department of Rehabilitation, Academic Medical Center Amsterdam, Amsterdam, The Netherlands; 3 Department of Psychology, Health and Technology, University of Twente, Enschede, The Netherlands; 4 Patient Association ALS Patients Connected, Rotterdam, The Netherlands; 5 Department of Neurology, Brain Center Rudolf Magnus, University Medical Center Utrecht, Utrecht, The Netherlands; 6 Department of Rehabilitation, Physical Therapy Science & Sports, Brain Center Rudolf Magnus, University Medical Center Utrecht, Utrecht University, Heidelberglaan 100, 3584 CX, Utrecht, The Netherlands

**Keywords:** Caregivers, Amyotrophic Lateral Sclerosis (ALS), Progressive Muscular Atrophy (PMA), Acceptance and Commitment Therapy, Psychological distress

## Abstract

**Background:**

Informal caregivers of patients with Amyotrophic Lateral Sclerosis (ALS) or Progressive Muscular Atrophy (PMA) face stressful demands due to severe impairments and prospect of early death of the patients they care for. Caregivers often experience feelings of psychological distress and caregiver burden, but supportive interventions are lacking. The objective of this study is to investigate the effectiveness of a psychosocial support program aimed at enhancing feelings of control over caregiving tasks and reducing psychological distress. This support program is based on an existing program for adult partners of people with cancer and is adapted to meet the needs of ALS caregivers.

**Methods:**

This study is a randomized controlled trial using a wait-list control design. One hundred and forty caregiver-patient dyads, recruited from a nationwide database and through the website of the Dutch ALS Center, will be either randomized to a support program or a wait-list control group. The blended intervention is based on Acceptance and Commitment Therapy and consists of 1 face-to-face contact, 6 online guided modules and 1 telephone contact. The intervention can be worked through in 8 weeks. The effectiveness and the participants’ satisfaction with the intervention will be evaluated using a mixed method design. Caregivers and patients will be asked to fill in questionnaires on 4 occasions during the study: baseline, 3 months, 6 months and 9 months. The main study outcome is the psychological distress of the caregiver assessed with the Hospital Anxiety and Depression Scale. Secondary outcomes are caregiver burden, caregiver quality of life, quality of life of the patient and psychological distress of the patient. Group differences in primary and secondary outcomes at 6 months will be compared with linear mixed model analysis. In a subgroup of caregivers we will explore experiences with the support program through semi-structured interviews. Usage of the online modules will be logged.

**Discussion:**

The study will provide insights into the effectiveness of a blended psychosocial support program on psychological distress of caregivers of patients with ALS or PMA, as well as into indirect relations with patients’ wellbeing.

**Trial registration:**

Netherlands Trial Registry NTR5734, registered 28 March 2016.

## Background

Informal caregivers, usually the patient’s partner, are key figures in Amyotrophic Lateral Sclerosis (ALS) and Progressive Muscular Atrophy (PMA) care. They provide the majority of support to patients and are often the primary caregivers. ALS and PMA are fatal motor neuron diseases, ALS affecting both upper and lower motor neurons while PMA only affects lower motor neurons. Informal caregivers of patients with ALS or PMA face stressful demands due to the prospect of an early death and severe impairments of the patient. Patients experience a progressive decline of muscle strength resulting in paralysis, difficulty with speech and swallowing, possible cognitive and behavioral problems and ultimately, respiratory failure leading to death [1–3]. Patients become increasingly impaired and the amount of care that is needed accumulates [4].

Since PMA is a rare subtype of motor neuron disease, research studies on PMA caregivers are limited. However, PMA shows substantial overlap with ALS and is considered to be a form of ALS [5]; PMA caregivers are, therefore, likely to struggle with the same issues as ALS caregivers. The wellbeing of ALS caregivers has been studied more intensively and shows that as the disease progresses, ALS caregivers experience heightened feelings of psychological distress and burden [6–8], which is related to a diminished quality of life [9]. The wellbeing of ALS caregivers is critical because a high level of burden might predict a breakdown in care, leading to earlier placement of the patient in a care-home or hospice [10].Therefore, improving the psychological health of the caregivers may not only improve their quality of life but also that of the patient.

Previous research has shown that psychological distress and feelings of burden of ALS caregivers are associated with disease characteristics of the patient (i.e. physical and behavioral problems) but also with characteristics of the caregivers themselves, such as their coping style or whether they find positive meaning in caregiving [7, 9, 11–15]. As the disease progresses, psychological and physical demands on the ALS caregiver increase. Patients become increasingly reliant on their caregiver, and caregivers have to take over responsibilities from the patient. Handling all these responsibilities, accepting a loved one’s illness and accepting the loss of the patient in the near future are examples of issues ALS caregivers struggle with [16].

ALS and PMA caregivers are faced with situations, yet may lack the relevant knowledge and skills, such as communicating about the disease and death, dealing with the patients’ behavioral changes, dealing with their own emotions or expressing their own boundaries [17]. Consequently, caregivers may not feel competent or in control with respect to their caregiving tasks, while the demands increase. From previous studies we know that a combination of high demands and feelings of insufficient control over caregiving is associated with poorer physical and psychological health outcomes of caregivers [18, 19]. Previous studies have indicated that there is a need for psychosocial interventions for caregivers, but such interventions are still lacking [6, 20, 21].

Acceptance and Commitment Therapy (ACT) is a form of cognitive behavioral therapy that encourages individuals to accept unwanted private events which are out of personal control (such as thoughts, feelings and memories) and to identify important values in life in order to engage in committed action to pursue these values [22]. The acceptance component in ACT makes this therapy valuable in contexts with circumstances that cannot be changed [23, 24], for instance, receiving a diagnosis of ALS or PMA. The values component supports caregivers to undertake action that is personally meaningful. This can assist them in adjusting to their situation, in moving on in life and in enhancing their psychological wellbeing [23]. Applying acceptance strategies and living up to personal values requires a different way of responding to situations and may increase the feeling of control [25].

ACT has proven to be effective in decreasing feelings of psychological distress in various target groups, including caregivers of other patient populations [26–28]. Recently, ACT has also been proved to be effective when delivered via the internet [28, 29]. Since ALS and PMA caregivers are often preoccupied with the care for their home-bound patient, receiving care in a more accessible and time efficient manner may offer opportunities.

In this study, we will investigate the effect of a blended support program in which face-to-face contact and e-health will be combined. The support program is based on Acceptance and Commitment principles and focuses particularly on the needs of ALS and PMA caregivers. This support program aims to diminish caregivers’ psychological distress by increasing their feelings of control in fulfilling the caregiving tasks for patients with ALS or PMA.

## Methods

The described protocol (Version 6, dated 27-07-2017) has been developed according to the Standard Protocol Items Recommendations for Interventional Trials (SPIRIT) and the Template for Intervention Description and Replication (TIDieR) [30, 31].

### Design

This study is a randomized controlled trial in which caregiver-patient dyads will be randomly allocated to one of two groups:

1. Intervention group (support program during 8–12 weeks).
2. Wait-list control group, receiving care as usual (6-month monitoring preceding the support program).

**Fig. 1:**
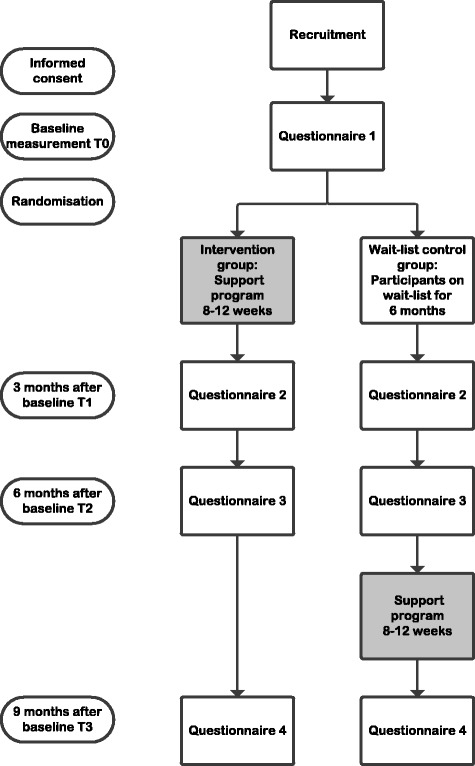
Flowchart study

This design enables us to investigate whether offering a support program in addition to usual care improves the wellbeing of caregivers compared to care as usual. Both caregiver and patient will be asked to complete online questionnaires at baseline (T0), 3 months after baseline (T1), 6 months after baseline (T2) and 9 months after baseline (T3), but only the caregivers will receive the support program. In Fig. 1, the flowchart of the study is presented. In a subgroup of caregivers, we will explore experiences with the support program through interviews.

### Study population

Caregiver-patient dyads will be recruited through a nationwide ALS/PMA database and via the website of the Dutch ALS Center. The study will also be announced on websites of ALS/PMA patient associations. Patient and inclusion criteria are 1) the caregiver is the partner of the ALS or PMA patient; 2) the caregiver is 18 years or older; 3) caregiver and patient are proficient in Dutch to fill out the questionnaires; 4) caregiver and patient have internet access. When patients are not able or not willing to complete online questionnaires, caregivers are still eligible to join the support program provided the patient consents. The inclusion criteria remain in force.

### Sample size

The sample size calculation was based on Hospital Anxiety Depression Scale (HADS) data of informal caregivers in a previously completed study [6]. The total number of caregiver-patient dyads needed to detect a clinically relevant difference [32] of 3.65 points, with a standard deviation of 7.3 between the groups at T2, with an alpha of 0.05 and a power of 80%, is 116 caregiver-patient dyads (58 dyads in each group). Taking into account an attrition rate of 20%, we aim to include 140 caregiver-patient dyads in total.

### Procedure

Caregiver-patient dyads recruited via the national ALS/PMA database will be contacted by telephone. In this telephone call, the dyad will receive information about the study. When dyads are interested in participating, the inclusion criteria will be checked. Eligible dyads receive the study information letter per postal mail. One week after sending the information letter, caregivers will be contacted by telephone. Caregivers who do not want to participate will be asked for their reasons for not participating and we will assess their burden of caregiving with the one item, Self-Rated Burden scale (SRB) [33].

Caregiver-patient dyads can find information about the research and can apply to participate on the Dutch ALS Center website. Thereafter, the researcher will send the research information letter. One week after sending the information letter, caregivers will be contacted by telephone to answer questions and to check the inclusion criteria.

Dyads are asked to return the signed informed consents by postal mail. Once the informed consents have been received, participants are sent an invitation via e-mail to fill out the first assessment (T0).

### Randomization

After completing the first assessment, dyads will be randomized into the wait-list control or experimental condition by the researchers according to a computerized programmed randomization scheme. Randomization will be stratified for the degree of functional impairment of the patient (Amyotrophic Lateral Sclerosis Functional Rating Scale-Revised, using the cut off score for severe disabilities ≥ 24) [34, 35], presence of behavioral problems of the patient (Amyotrophic Lateral Sclerosis-Frontotemporal Dementia-Questionnaire, using the cut off score for mild behavioral changes ≥ 22) [36] and gender of the caregiver.

### The intervention

The content of the support program for informal caregivers is based on an online intervention aimed at partners of patients with cancer [37, 38]. This intervention, based on ACT, was adapted to the specific needs of ALS caregivers. We interviewed 21 ALS caregivers about their support needs [17] and added information and exercises related to these needs to the support program. Next, we asked 6 ALS caregivers and professionals in ALS/PMA care and research (physicians, psychologists and researchers) to provide feedback on the content of the program. Based on their feedback, text materials were adjusted and the web-based application was developed. In a usability test, five partners were observed while using the web-based application and they were asked to evaluate it. Their feedback was used to improve the usability of the web-based application.

The support program consists of an introductory face-to-face appointment with a psychologist, 6 psychologist-guided online modules and one closing telephone contact with the psychologist. The total program can be completed in 8 weeks. If caregivers need more time due to personal circumstances, they have the opportunity to work through the total program in 12 weeks. The content of the support program, the topics and the goals of each part are presented in Table 1.

**Table 1:** Content intervention

Part of intervention	Topics	Goals	Key components
Face-to-face session	• The care situation • Wellbeing caregiver • Information about support program • Log in online modules	To receive information about the care situation and establish a relationship between the psychologist and the caregiver. To inform about caregiver burden and start online modules.	• Psychoeducation
Online module 1 Coping with your emotions and thoughts	• Dealing with and expressing emotions • Recognizing thoughts	To recognize emotions and encourage caregivers to allow, express and share emotions that can arise. To recognize dysfunctional thoughts and rumination. Change the way the caregiver relates to thoughts/to create distance from thoughts.	• Acceptance • Cognitive defusion • Mindfulness
Online module 2 The art of communication	• Communication style • Communication about sensitive topics • Communication about providing care	To improve the overall communication and to communicate with the patient about sensitive topics and providing care in the future.	• Communicating about what really matters • Mindfulness
Online module 3 Your resilience plan	• Dealing with continuous stress • Moments of relaxation • Using your sources	To make a resilience plan that may allow caregivers to maintain health during this stressful period by taking care of themselves.	• Acceptance • Mindfulness
Online module 4 What is really important	• Values in relationship • Values in life	To identify the values of the caregiver in different areas of life and to plan action to meet these values.	• Values • Commited action • Mindfulness
Online module 5 Moments of joy	• Positivity during difficult times • Celebrate the relationship	To seek, enjoy and cherish the positive moments in the relationship and in life.	• Committed action • Mindfulness
Online module 6 A good last period	• Life story of the patient • Communication in this last phase • Beautiful memories • Being grateful	To create a beautiful last period with the loved ones and to make memories with the patient for the future.	• Acceptance • Communicating about what really matters • Committed action • Mindfulness
Telephone call	• Any questions • Finish the support program	To offer support with regard to any issues and close the support program.	

#### Face-to-face session

Participants will have a one-hour session with a psychologist before they start with the online modules at the residence of the caregiver. The session is protocolized: the psychologist briefly explains the purpose of the intervention, receives information about the caregiving situation, logs in and demonstrates the online program and establishes a working relationship with the participant. Following this session, the caregiver starts with the online modules.

#### Online modules

The online part consists of 6 online modules, each module is directed at a specific theme. All modules start with an introduction directed at the theme of the module, followed by psychological exercises. The content of the online modules is focused on the following key components: 1] acceptance (embracing the private events without unnecessary attempts to change them [39]), 2] values (identifying valued domains of life [40]), 3] committed action (actions to pursue one’s values [40]), 4] mindfulness (training conscious awareness and attention from one moment to the next moment [41]), 5] communication about what really matters, 6] cognitive defusion (change the way one interacts with or relates to thoughts by altering the contexts in which they occur [39]). Participants also receive practical information, tips and references to relevant websites, organizations and other sources of information and support associated with the theme of the module. They are able to get in contact with other participating caregivers of patients with ALS or PMA, using the online program. They have an online personal profile and can send each other private messages. Participants can also share tips and advice with fellow participants.

The same psychologist who visited the participant for the face-to-face session provides online feedback including feedback on the completed exercises, a reflection on the progress of the participant and a reaction to any questions or difficulties.

#### Telephone contact

The program ends with a telephone call with the psychologist. During this call, the caregiver can ask for advice for specific problems and discuss questions that came up after completing the last module.

#### Guidance

The support will be provided by psychologists who are trained to provide the intervention.

### Assessments

All quantitative assessments are self-report measures and will be administered online. Overviews of the questionnaires for caregivers and patients and their time of assessment are provided in Tables 2 and 3. Participants who discontinue the intervention will be asked to complete study follow-up assessments. Semi-structured qualitative interviews will be conducted by telephone.

**Table 2:** Measurement overview caregivers

		Measurement^a^			
Outcome	Instrument	T0	T1	T2	T3
*Socio-demographics*					
Caregiver, patient, and care characteristics	iMTA Valuation of Informal Care Questionnaire	x			
*Primary outcome*					
Psychological distress	Hospital Anxiety and Depression Scale	x	x	x	x
*Secondary outcomes*					
Quality of life	Care-related Quality of Life −7 + Care-related Quality of Life - VAS	x	x	x	x
Burden	Zarit Burden Interview + Self-Rated Burden scale	x	x	x	x
*Mediator*					
Self-efficacy	Revised scale for caregiving self-efficacy	x	x	x	x
*Covariates*					
Satisfaction with relationship	Satisfaction Questionnaire	x	x	x	x
Social Support	Multidimensional Scale of Perceived Social Support	x	x	x	x
Behavioral changes patient	Amyotrophic Lateral Sclerosis-Frontotemporal Dementia- Questionnaire	x	x	x	x
Physical functioning patient	Amyotrophic Lateral Sclerosis Functional Rating Scale- Revised	x	x	x	x
*Evaluation*					
Evaluation intervention	Client Satisfaction Questionnaire + Self developed scale		x^b^		x^c^

^a^T0 = Baseline, T1 = 3 months, T2 = 6 months, T3 = 9 months

^b^only for the intervention group

^c^only for the wait-list control group

**Table 3:** Measurement overview patients

		Measurement^a^			
Outcome	Instrument	T0	T1	T2	T3
*Secondary outcomes*					
Quality of life	McGill Single Item Scale	x	x	x	x
Psychological distress	Hospital Anxiety and Depression Scale	x	x	x	x
*Covariates*					
Self-perceived as burden	Self-perceived Burden Scale - 1 item	x	x	x	x

^a^T0 = Baseline, T1 = 3 months, T2 = 6 months, T3 = 9 months

#### Primary outcomes measure

##### Caregivers’ psychological distress

Psychological distress will be measured using the HADS [42, 43]. This scale consists of 14 items reflecting symptoms of anxiety and depression by 7 items each. Items are scored on a 4-point scale and total scores range from 0 to 42. Furthermore, a total score for the subscales depression and anxiety can be calculated. The internal consistency for the total scale and both subscales is sufficient to high (Cronbach’s alpha ranging from .71 to .90). The test-retest reliability for the total scale and both subscales proved to be high (correlation coefficient ranging from .86 to .91) [42, 44].

#### Secondary outcomes measures

##### Caregiver burden

The Zarit Burden Interview (ZBI) will be used to measure caregiver burden by evaluating disease impact on caregivers’ quality of life, psychological suffering and impact on social and family relationships [45]. We will use a short version of 12 items, which has shown to have comparable psychometric properties to the full version that consists of 22 items [46]. The ZBI contains a 0–4 point scoring system with the following answering options: never, rarely, sometimes, quite frequently and nearly always. The questionnaire yields a maximum score of 48. A score ≥ 17 indicates a high burden. The ZBI short form shows good validity, internal consistency, and discriminative ability [47].

Burden of caregiving will additionally be measured with the Self-Rated Burden scale (SRB) [33]. The SRB is a single question in which informal caregivers are asked to give an overall assessment of the burden they experience from caring by using a visual analogue scale. The scores range between ‘0’ (not at all straining) and ‘10’ (much too straining). The SRB is a valid and reliable question and it can be used for a quick screening of caregivers at risk [33].

##### Caregivers' quality of life

Caregivers’ quality of life will be assessed using the Care-related Quality of Life (CarerQoL) [48]. The CarerQoL combines a description of the burden of caregiving on seven care dimensions (CarerQoL-7) with a valuation component (CarerQoL-VAS) assessing general quality of life in terms of happiness. The CarerQoL-7 provides answering categories ‘none’ (1), ‘some’ (2), and ‘many’ (3). The CarerQoL-VAS contains 0, ‘completely unhappy’ and 10 ‘completely happy’ as endpoints. The psychometric properties of the CarerQol were shown to be satisfactory [48–50].

##### Patients’ quality of life

Patients’ self-rated quality of life will be measured using the McGill Quality of Life Questionnaire (MQOL) [51], which is designed to measure the quality of life of patients with a terminal illness. In order to burden the patients as little as possible, we will only use the single item scale which assesses the overall quality of life with answer scores ranging from 0 = very bad to 10 = excellent.

##### Patients’ psychological distress

Patients’ psychological distress will be measured with the HADS.

#### Mediator

##### Caregivers’ self-efficacy

Caregivers’ beliefs about their capacity to carry out caregiving tasks will be measured using the Revised Scale for Caregiving Self-Efficacy [52]. The original version of the instrument consisted of 15 items within 3 subscales; self-efficacy for obtaining respite, responding to disruptive patient behaviors, and controlling upsetting thoughts about caregiving. The disruptive patient behaviors scale is not suitable for our target population and is omitted.

Caregivers are asked to indicate on a scale of 0 (absolutely cannot do) to 100 (certainly can do) how confident they are with respect to items such as “how confident are you that you can control worrying about future problems that might come up with [patient]”. All subscales demonstrate strong internal consistency and adequate test-retest reliability [52]. We added 3 additional questions based on the Job Content Questionnaire aimed at the control that caregivers perceive over fulfilling the caregiver tasks [53].

#### Covariates

##### Caregivers’ social support

Caregivers’ experience of social support will be measured using the Multidimensional Scale of Perceived Social Support (MPSS) [54]. The MPSS consists of 12 items and is aimed at different sources of social support (family, friends, and significant others). The items are scored on a 7-point Likert scale ranging from 1 (very strongly disagree) to 7 (very strongly agree). The total score is calculated by adding up the scores of all items, resulting in a range of 12–84. A higher score indicates stronger social support, with scores ≥ 79 corresponding to an experience of strong support. MPSS has proven to be a psychometrically valid instrument, with good test-retest reliability and adequate validity among varying populations [54–56].

##### Caregivers’ satisfaction with relationship

Caregivers’ satisfaction with the relationship with the patient will be assessed using the Satisfaction Scale [57]. The questionnaire consists of 4 satisfaction items which are rated on a scale ranging from 1 (not satisfied) to 5 (satisfied). A total score is calculated by adding up the scores of the 4 items, with a higher score indicating more satisfaction with the relationship. The items refer to caregivers’ experience during the last month. The satisfaction scale shows reasonable internal consistency [58].

##### Patients’ behavioral changes

Behavioral changes in patients will be assessed with the Amyotrophic Lateral Sclerosis-Frontotemporal Dementia-Questionnaire (ALS-FTD-Q) [36]. The questionnaire asks the caregiver to compare the patient’s current behavior with his/her behavior 3 years ago. It consists of 25 items with a total score range of 0–100 (≥ 22 indicating mild behavioral changes and ≥ 29 corresponding to significant behavioral changes). The ALS-FTD-Q shows good internal consistency (Cronbach’s  alpha = 0.92) as well as construct validity [36].

##### Patients’ physical functioning

The physical functioning of patients will be assessed using the Amyotrophic Lateral Sclerosis Functional Rating Scale-Revised (ALS-FRS-R) [35]. The scale consists of 12 items with 0–4 point scores in order to measure limb, bulbar, and respiratory dysfunction. An example is the item “Walking” with answer scores 0 = normal to 4 = is unable to consciously move legs. Overall scores range from 0 to 48, with higher scores indicating better physical functioning. The ALS-FRS-R demonstrates strong internal consistency as well as construct validity [35]. This questionnaire will be completed by the caregiver.

##### Patients’ perception of being a burden

Patient’s own feelings of being a burden for the caregiver will be measured using one item of the Self Perceived Burden Scale (SPBS): “I feel that I am a burden to my caregiver” [59]. This statement is rated on a scale of how often patients feel this way, from “none of the time” (1) to “all of the time” (5). Higher scores indicate that the patients perceived themselves to cause a higher burden to their caregivers.

#### Evaluation of the intervention

##### Satisfaction with received support

To measure the satisfaction of the caregiver for the support they received, the 8-item Client Satisfaction Questionnaire (CSQ-8) is used [60]. All items are scored on a 4-point scale ranging from 1 to 4. Response options differ from item to item. An example is “How satisfied are you with the amount of help you have received?” (for which the response options range from 1 = “Quite dissatisfied” to 4 = “Very satisfied”). An overall score is calculated by summing and ranges from 8 to 32, with higher scores indicating greater satisfaction. The Dutch translated version of the questionnaire shows high internal consistency (.91) [61].

##### Evaluation support program

Additionally, a scale to evaluate the intervention was developed. The participant is asked to rate the intervention in general and the different components of the intervention such as the psychological exercises, contact with the psychologist who provided the feedback and contact with other informal caregivers. Participants are asked to rate every component on a 0–10 scale; the questionnaire consists of 9 questions.

##### Experiences with support program

Semi-structured interviews to explore the experiences of the caregivers with the support program will be carried out after subjects complete the support program. Participants will be selected via purposive sampling on demographic variables (age, sex, disease stage patient). Interviews will be held by a researcher, using a topic list with the following topics: experiences with support program, user-friendliness, use of the support program, valuable, missing and redundant elements of the support program and recommendations for change. The interviews will last approximately 1 h and will be recorded. Participants will be included until data saturation is reached.

#### Demographics and description of the care situation

##### Demographics and care situation

The iMTA Valuation of Informal Care Questionnaire (iVICQ) is a questionnaire which facilitates an accurate description of providing informal care and its effects on informal caregivers [62]. We used the sections of the background information of patients and caregivers, the informal care situation and questions to economically validate informal care as a directory for our questions regarding these subjects.

#### Questions to assess the working mechanism of the support program

The support program aims to improve feelings of control over caregiving and reduce psychological distress. Therefore, at the end of every online module, the caregiver is asked two questions about “feelings of control over executing caregiving tasks” and “the level of distress they experience”, at that moment on a VAS scale [63].

#### Monitoring adherence to the intervention modules

In order to assess the use of and the adherence to the online modules we will collect log data of the participants such as the frequency of logging in, the duration of logging in, which parts of the modules are downloaded and which functions are used.

### Data management

All personal data will be coded, removed from the data for analysis and stored separately. Only designated research staff will have access to the keys linking the data with the personal information. The research team will have access to the final dataset. Data management and monitoring of the trial will be performed by qualified personnel according to standard operation procedures of the Brain Center Rudolf Magnus, University Medical Center Utrecht.

### Analyses

#### Statistical analyses

Descriptive statistics will be used to report demographic variables, clinical outcomes, and the use of the different modules. Group differences in primary and secondary outcomes will be compared with linear mixed model analyses, in which the mediator and covariates will be included. Statistical analyses will be performed primarily according to intention-to-treat and secondarily according to per-protocol principles. The intention to treat analyses will include data of all included caregivers, regardless of their adherence to the intervention or their missing data. In the per-protocol analyses we will only include caregivers who completed at least 4 modules (66.7%) and the T2 measurement. All hypotheses will be tested 2-sided, with a critical value of 0.05. Effect sizes on the primary outcome variable (HADS total) will be calculated with Cohen’s D using the means and pooled standard deviations of the two groups.

#### Interview analyses

Interviews will be transcribed and analyzed thematically [64]. The texts will be broken down into fragments based on content and fragments will be labeled with a code using NVIVO 10 [65, 66]. Once the coding of all interviews has been completed, codes will be sorted according to similarities and overarching themes and subthemes will be identified.

## Discussion

To our knowledge, this will be the first study to evaluate a blended support program for caregivers of ALS and PMA patients. The program is aimed at enhancing feelings of control over caregiving tasks using ACT principles. Previous research on ALS caregiving revealed increasing levels of psychological distress in caregivers, a lack of existing interventions and an urgent need for support [7, 21]. ACT interventions have proven to reduce psychological distress in other caregiver populations [27, 28] and are valuable in contexts with circumstances that cannot be changed [23, 24].

A strength of this intervention is the blended approach: face-to-face support in combination with online support. Due to the many hours ALS and PMA caregivers spend on providing care, they often experience a lack of personal time [17], which reduces the opportunity to access traditional forms of support. Therefore, the blended approach may provide support in a more time-efficient manner, as caregivers can access information and exercises any time at home via an online platform.

Although previous research has provided information on factors associated with psychological distress and burden [e.g. 15], the underlying process is still unclear due to a gap in research on personal factors related to the caregiver. The use of a theoretical framework is considered as another strength since it helps to gain insight into whether the demand-control theory is applicable to the caregiver situation in ALS and PMA [18] and will provide knowledge on the influence of factors such as control and mastery in relation to psychological distress and burden. This will provide information to understand how, when and for whom the intervention will be effective.

Further, caregivers and healthcare professionals were involved in both the development process of the intervention and the design of the study. Due to their involvement, we were able to develop an intervention that meets the needs and wishes of caregivers and includes the most important themes according to professionals. Caregivers and professionals will also be involved in the next steps of the research such as the recruitment and the dissemination of the results of the study. Previous studies indicated that engaging the target group increases study enrollment and may enhance the uptake and the acceptance of interventions [67, 68].

Another strength of the study design is the mixed method approach; questionnaires and interviews will be used to evaluate the program which enables a throughout evaluation and may lead to further improvement of the support program.

The support program may also have some weaknesses. First of all, the online part of the support program might be an obstacle for some caregivers due to a lack of information and communication technology literacy. These caregivers might prefer to receive traditional face-to-face support.

Another limitation might be that the intervention is only focused on partners, which means that primary caregivers who have another type of relationship with the patient are excluded while they might be in need of support. Once the intervention has proven to be effective, it might be worthwhile developing an adapted version for primary caregivers with other relationships to the patient.

A limitation of the study design might also be contamination with care as usual. In the last couple of years, the value and the importance of the social environment of patients has been emphasized. This has led to a stronger focus on caregivers in standard care; care facilities are encouraged to involve caregivers in their care plans. Due to this recent shift, the support for caregivers might have improved and it may be more difficult to demonstrate a significant difference when we compare care as usual with our support program. However, if caregivers perceive the support program as being more user-friendly than care as usual, this will encourage its use in standard care.

To conclude, this study will provide insight into the effects of a blended support program for informal caregivers of patients with ALS and PMA by targeting feelings of control over caregiving tasks using ACT principles. The program could potentially benefit caregivers, and might affect patients’ wellbeing indirectly.

### Dissemination plan

Results of this study will be published in international, peer-reviewed journals and presented at relevant conferences/congresses, both national as well as international. Results will furthermore be communicated through national publications and published on relevant websites.

## Abbreviations

ACT: Acceptance and Commitment Therapy
ALS: Amyotrophic Lateral Sclerosis
ALS-FRS-R: Amyotrophic Lateral Sclerosis Functional Rating Scale-Revised
ALS-FTD-Q: Amyotrophic Lateral Sclerosis-Frontotemporal Dementia-Questionnaire
CarerQoL: Care Related- Quality of Life
CSQ-8: 8-item Client Satisfaction Questionnaire
HADS: Hospital Anxiety and Depression Scale
iVICQ: institute for Medical Technology Assessment Valuation of Informal Care Questionnaire
MPSS: Multidimensional Scale of Perceived Social Support
MQOL: McGill Quality of Life Questionnaire
PMA: Progressive Muscular Atrophy
SPBS: Self Perceived Burden Scale
SPIRIT: Standard Protocol Items Recommendations for Interventional Trials
SRB: Self-Rated Burden scale
TIDieR: Template for Intervention Description and Replication
ZBI: Zarit Burden Interview
